# Stochastic Inheritance of Division and Death Times Determines the Size and Phenotype of CD8^+^ T Cell Families

**DOI:** 10.3389/fimmu.2019.00436

**Published:** 2019-03-14

**Authors:** Aridaman Pandit, Rob J. De Boer

**Affiliations:** ^1^Laboratory of Translational Immunology, University Medical Center Utrecht, Utrecht University, Utrecht, Netherlands; ^2^Department of Rheumatology and Clinical Immunology, University Medical Center Utrecht, Utrecht University, Utrecht, Netherlands; ^3^Theoretical Biology and Bioinformatics, Utrecht University, Utrecht, Netherlands

**Keywords:** CD8^+^ T cells, single cell dynamics, stochastic inheritance, T cell differentiation, immunological memory

## Abstract

After antigen stimulation cognate naïve CD8^+^ T cells undergo rapid proliferation and ultimately their progeny differentiates into short-lived effectors and longer-lived memory T cells. Although the expansion of individual cells is very heterogeneous, the kinetics are reproducible at the level of the total population of cognate cells. After the expansion phase, the population contracts, and if antigen is cleared, a population of memory T cells remains behind. Different markers like CD62L, CD27, and KLRG1 have been used to define several T cell subsets (or cell fates) developing from individual naïve CD8^+^ T cells during the expansion phase. Growing evidence from high-throughput experiments, like single cell RNA sequencing, epigenetic profiling, and lineage tracing, highlights the need to model this differentiation process at the level of single cells. We model CD8^+^ T cell proliferation and differentiation as a competitive process between the division and death probabilities of individual cells (like in the Cyton model). We use an extended form of the Cyton model in which daughter cells inherit the division and death times from their mother cell in a stochastic manner (using lognormal distributions). We show that this stochastic model reproduces the dynamics of CD8^+^ T cells both at the population and at the single cell level. Modeling the expression of the CD62L, CD27, and KLRG1 markers of each individual cell, we find agreement with the changing phenotypic distributions of these markers in single cell RNA sequencing data. Retrospectively re-defining conventional T-cell subsets by “gating” on these markers, we find agreement with published population data, without having to assume that these subsets have different properties, i.e., correspond to different fates.

## 1. Introduction

The hallmark of adaptive immunity is its ability to mount a specific response against primary infection and to rapidly respond against re-infections with an enlarged population of memory cells. This immunological memory forms the basis of vaccination. CD8^+^ T cells mount cytotoxic responses against intracellular pathogens like viruses and form an integral part of the adaptive immune system. CD8^+^ T cells express a T cell receptor (TCR) allowing them to recognize their cognate antigen presented as a pMHC complex (1–3). Naïve CD8^+^ T cell are activated when their TCRs bind a cognate pMHC complex, and after tandem signaling by co-stimulatory molecules. After activation naïve CD8^+^ T cells undergo clonal expansion producing short-lived effector and longer-lived memory cells. For a typical epitope in mice, there are less than a 1,000 cognate naïve CD8^+^ T cells, which expand and can produce a progeny of more than 10^7^ activated cells (4, 5). When antigen is cleared the CD8^+^ T cell population contracts about a 20-fold, leaving behind a long-lived population of memory T cells. These memory CD8^+^ T cells can rapidly respond upon secondary infection. The timing and magnitude of CD8^+^ T cell responses varies for different antigens and infections, but for each antigen the dynamics of the entire response is highly reproducible (6–9).

In contrast to the reproducible population dynamics, single-cell tracing studies have shown that the fate of individual naïve CD8^+^ T cell is heterogeneous (8, 10). Multiple lines of evidence suggest that the fate of an individual CD8^+^ T cell is regulated by their local niche, i.e., the cytokines, cell-cell interactions, co-stimulatory molecules, strength of TCR binding, and cell migration (6, 7, 9, 11–14). Lineage tracing studies have shown that individual naïve CD8^+^ T cell have the potential to produce both effector and memory T cells (6). Even in controlled *in vitro* experiments, genetically identical naïve CD8^+^ T cells expand into heterogenous “families” (15–17). Because several biological factors govern the fate of individual cells, this calls for involving stochasticity when modeling T cell differentiation.

Different experimental and mathematical models considering linear or branched differentiation pathways have been used to study the potential mechanisms of T cell differentiation and memory formation (7, 9, 18). According to the *effector first models* supported by epigenetic studies, naïve CD8^+^ T cells first divide and differentiate into effector cells during the expansion phase, which either die or differentiate into memory CD8^+^ T cells during the contraction phase (19–23). According to the *progressive differentiation models*, individual naïve CD8^+^ T cells receiving ample stimulation differentiate into effector cells, while those receiving less stimulation differentiate into memory cells (7, 9, 13). According to the *asymmetric division model*, naïve CD8^+^ T cells divide asymmetrically producing one daughter with effector potential, and another with memory potential (24). Thus, very different models have been proposed for CD8^+^ T cell differentiation.

Two single-cell tracing studies demonstrated a large heterogeneity in the number of progeny produced by individual naïve CD8^+^ T cells expressing the same TCR (8, 10). Buchholz et al. (10) compartmentalized the cells using the surface expression of the CD62L and CD27 markers, and found that the progeny (or family) of an individual naïve CD8^+^ T cell was also heterogenous in the fraction of “central memory” (CD62L^+^CD27^+^), “effector memory" (CD62L^−^CD27^+^), and “effector” (CD62L^−^CD27^−^) T cells. Thus, considering the surface expression of CD62L and CD27 as a marker for the fate adopted by individual cells, (10) found that individual naïve CD8^+^ T cells have very different memory potentials. Using the time course of these markers, Buchholz et al. (10) show that a progressive differentiation model from naïve to central memory, to effector memory, to effector cells fitted their single-cell tracing data best. One of the drawbacks of using molecules like CD62L and CD27 as a memory marker is that these molecules are also expressed on naïve CD8^+^ T cells. Recently activated T cells that are on a trajectory to become effector T cells are therefore expected to initially exhibit the CD62L^+^CD27^+^ central memory phenotype. Indeed, in both CD4^+^ (14) and CD8^+^ (25, 26) T cells, CD62L expression declines during the first division(s), but is either retained or up-regulated later in a sub-population of cells that one typically associates with memory precursor cells.

Division and differentiation of CD8^+^ T cells is a complex process. The growth of single-cell sequencing technologies and other high-throughput methods, including cellular barcoding and lineage tracing, warrants the development of models that can incorporate the complex dynamics of individual cells, along with their RNA/protein expression data. We modeled CD8^+^ T cell division and differentiation dynamics as competition between division and death fate of a cell, as advocated in the Cyton model (27). The same group of authors also developed models where daughter cells inherit these division and death times from their mother cell in stochastic manner (27–30). We here develop a simple combination of both models by defining a phenomenological stochastic inheritance of the division and death times (using lognormal distributions), in order to study the heterogeneity between the families produced by single naïve T cells. To integrate these growth dynamics with the molecular data, we also allow each cell to inherit three surface markers from their mother cell (i.e., CD62L, CD27, and KLRG1). Because cell division is often associated with differentiation (21, 27, 29, 31–33), we considered that the marker expression of a cell changes with cell division, without *a priori* coupling marker expression to the kinetic properties, or fate, of that cell. We show that such simple “stochastic inheritance” models can qualitatively replicate previously observed CD8^+^ T cell division and differentiation dynamics (10), both at the population level and at the single-cell level. Additionally, this stochastic inheritance of surface markers can account for the recent single-cell expression data obtained during the expansion phase of CD8^+^ T cells (26). Since in our model the expression of the markers on a cell has no effect on its kinetic properties, and the model nevertheless remains in agreement with the data, we conclude that compartmentalizing dividing T cells into kinetically different T cell subsets on the basis of their surface markers need not capture the true population dynamics, nor the fate adopted by individual T cells.

## 2. Results

### 2.1. Basic Model

**Figure 1:**
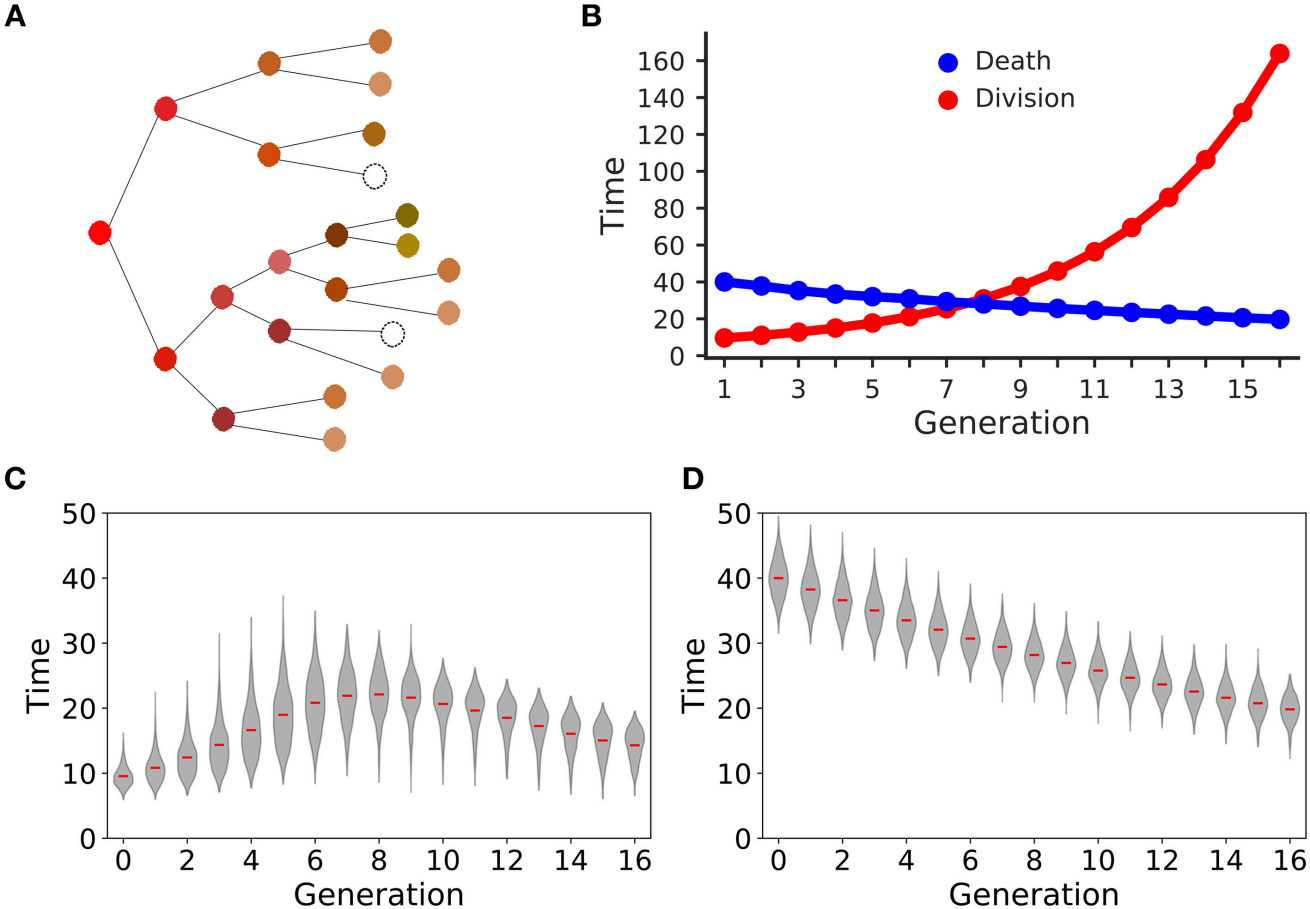
Stochastic inheritance model of T cell differentiation. Panel **(A)** is a schematic representation of the model. The red circle on the left represents a mother naïve CD8^+^ T cell that divides into two daughter T cells, which divide further. The color of the circles indicates that the dividing CD8^+^ T cells differ from each other in terms of their division and death kinetics. Empty circles represent dead cells. Panel **(B)** depicts that we parameterized the model such that expected division and death times cross each other at generation 8. The red line represents the expected mean division times at each generation and the blue line represents the expected mean death times at each generation. The violin plot shows the time of division **(C)** and death **(D)** distributions for each generation of dividing CD8^+^ T cells. The red lines depict the mean of each distribution. The model was initialized with a 1,000 naïve CD8^+^ T cells.

We simulated 8 days of clonal expansion of CD8^+^ T cells using a “stochastic inheritance” model (see Figure 1A and Table 1). The simulations were initialized with a 1, 000 naïve CD8^+^ T cells, and each cell was assigned a time of division (*t*_*p*_) and a time of death (*t*_*d*_), following earlier models (27–30). In our model, these “waiting times” are sampled from two independent lognormal distributions (Equations 1, 4). In generation zero the mean time of division was much lower than the mean initial time of death (see Figures 1B–D; Equation 1) to allow for an initial expansion phase. In our basic model, we considered that all naïve CD8^+^ T cells become activated at the start of the simulation, and at their time of division these cells deliver two daughter cells. Each daughter cell noisily inherits the division and death times from its mother cell (Equations 2, 3). Since the probability of cell death has been shown to increase with the number of divisions, we decrease the probability to undergo a subsequent division (i.e., we increase the division time; see Equation 2), and increase the probability of cell death (i.e., we decrease the death time; see Equation 5), with every division. The inheritance of the division and death times is stochastic because we add noise to the time both daughters inherit from their mother (using a lognormal distribution), and add Gaussian noise to allow for some variation between the daughters. Thus, one daughter cell may divide or die earlier than the other, but their division and death times are correlated to those of their mother (Figure 1A).

**Table 1:** Parameter values used in the stochastic inheritance model.

**Parameter**	**Value**	**Dimension**
*μ*_*mp*_	4.5	hours
*v*_*mp*_	0.2 × *μ*_*mp*_	hours
*μ*_*p*_	1.25	–
*v*_*p*_	0.05 × *μ*_*p*_	–
*t*_*min*_	4.0	hours
*v*_*e*_	0.02	–
*μ*_*md*_	40	hours
σ_*md*_	0.2 × *μ*_*md*_	hours
*μ*_*d*_	0.99	–
σ_*d*_	0.05 × *μ*_*d*_	–

Figure 1C shows that the mean division time of the proliferating cells increases with every division for the first 8 divisions, and, surprisingly, starts to decrease after 8 divisions (even though in our model the probability to divide decreases with every division). Since the probability of cell death increases with every division, the death time is expected to become comparable to the division time (Figures 1B,D) after 8 divisions, resulting in preferential death of slowly dividing CD8^+^ T cells. Although the increased death rate slows down the expansion of the population, there is a selection for cells that divide fast, which decreases the mean division time after about 8 divisions. We parameterized our model such that the mean division and death times become comparable at 8th generation (Figure 1B); choosing different parameters did not alter our subsequent results qualitatively (Figure S1 in Supplementary Material). Thus, modeling time of division and death as independent stochastic variables allows for complex population dynamics selecting for particular cellular properties in the absence of competition between the cells.

#### 2.1.1. Disparate Single Cell Behavior

**Figure 2:**
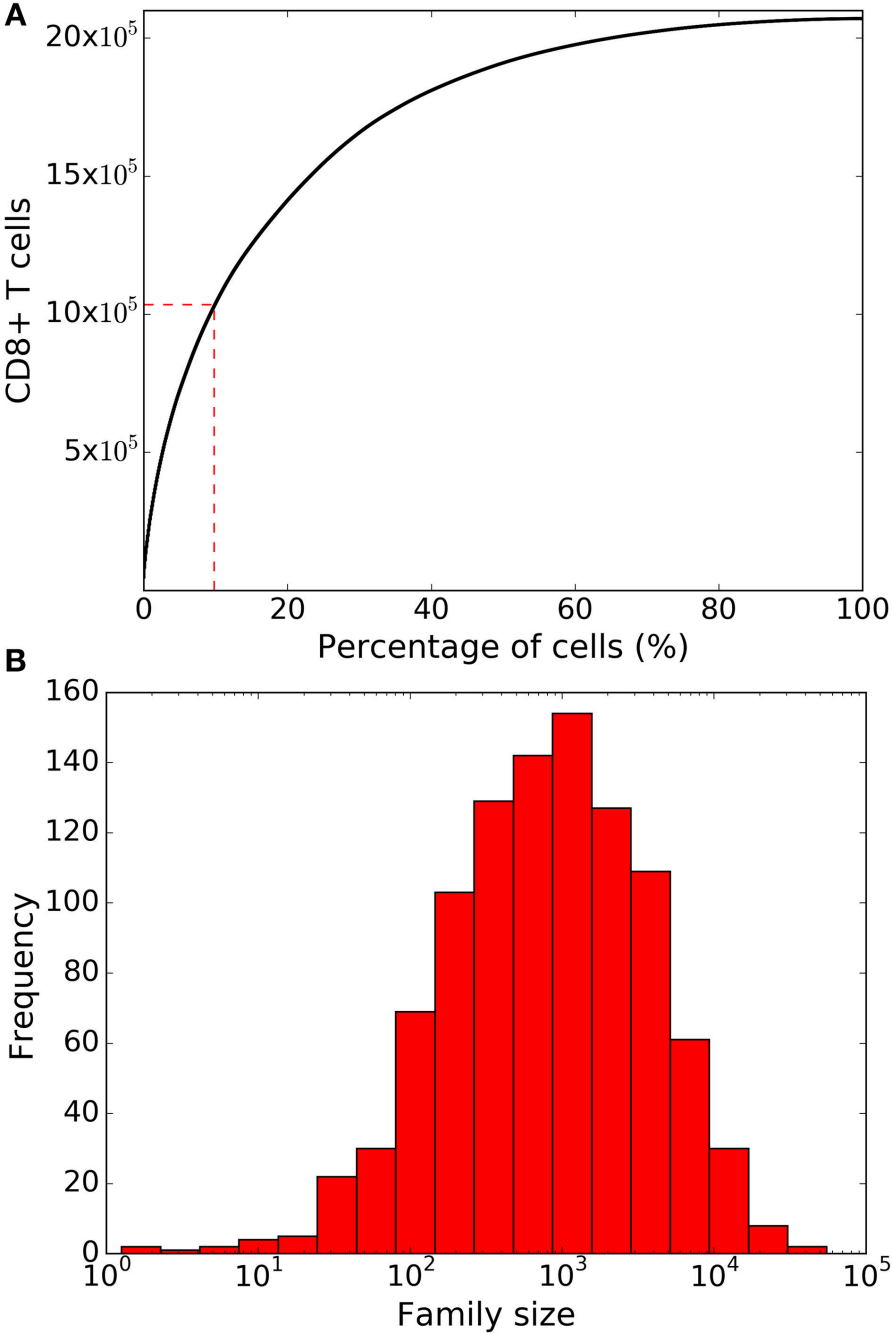
Family size distributions generated by the basic stochastic inheritance model. Panel **(A)** depicts the cumulative family size distribution, i.e., the percentage of naïve CD8^+^ T cells (x-axis) and the corresponding cumulative number of CD8^+^ T cells present on day 8 (y-axis). The CD8^+^ T cell families on the x-axis are ordered by their size on day 8 (with the largest family on the left). The dashed red lines represent the fraction of families that constitute 50% of the total response. Panel **(B)** shows the frequency distribution of family sizes on day 8, which reveals that some families contribute more than 10^4^ cells to the total response. These results are based on simulating the clonal expansion of a 1,000 naïve CD8^+^ T cells.

After 8 days of clonal expansion, our simulations typically produced more than 10^6^ CD8^+^ T cells starting from a 1,000 naïve CD8^+^ T cells. Previous studies have shown that individual naïve CD8^+^ T cells carrying the same T cell receptor make disparate immune responses (8, 10). In good agreement with the experimental studies (4, 8, 10), we found that a small fraction of the initial naïve CD8^+^ T cell pool form the majority of the response (Figure 2A). Specifically, about 10% of the naïve CD8^+^ T cells contribute about 50% of the total immune response. In the model this disparity can only arise from the fact that different CD8^+^ T cells families undergo different numbers of divisions. In a typical simulation, the median family size (i.e., the number of progeny of a single naïve CD8^+^ T cell) was about a 1000 cells, whereas the largest family size was about 10^5^ cells (Figure 2B). Thus, stochastic inheritance of division and death times can account for the experimentally observed large disparity in family sizes.

### 2.2. Surface Marker Distribution

**Figure 3:**
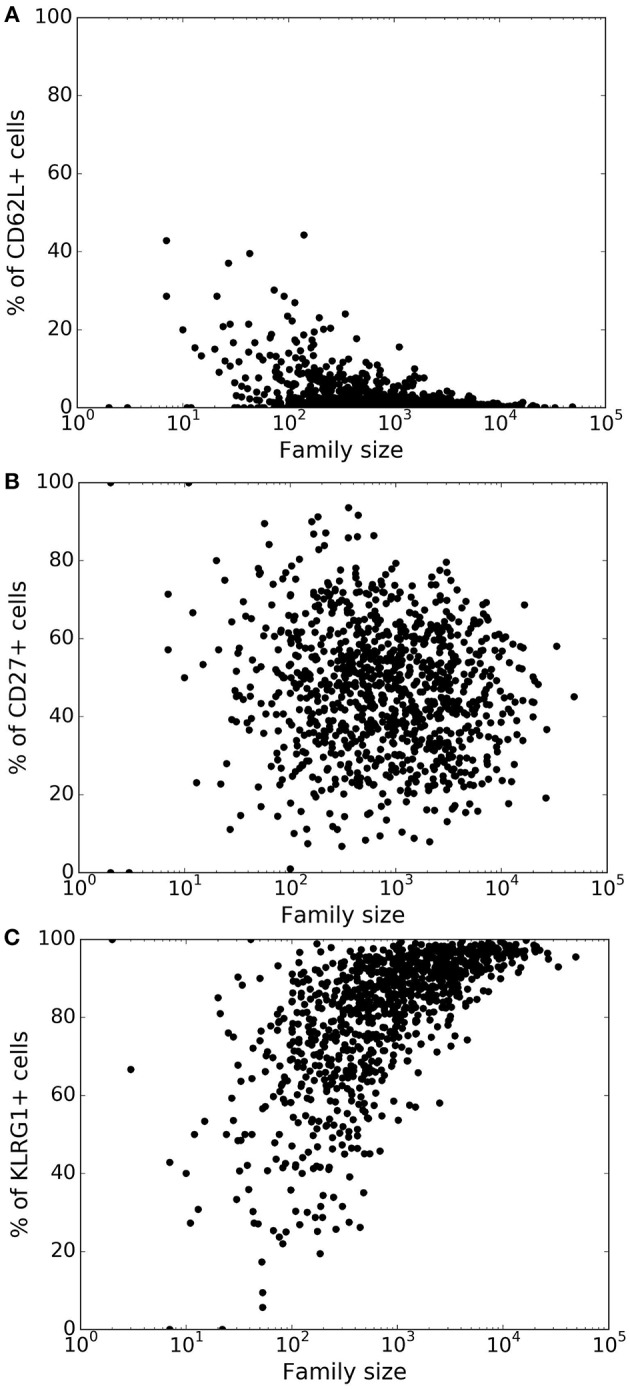
Variation of the marker distribution as a function of family size in the basic model. The fraction of CD8^+^ T cells that are positive for CD62L **(A)**, CD27 **(B)**, and KLRG1 **(C)** markers is shown for each CD8^+^ T cell family. Each bullet represents one naïve CD8^+^ T cell family. These results are based on simulating the clonal expansion of a 1,000 naïve CD8^+^ T cells.

Lineage tracing experiments have used several surface markers, including CD62L, CD27, and KLRG1, to categorize CD8^+^ T cells as naïve, central memory, effector memory and effector T cell subsets (8, 10, 26). To study marker dynamics, we allowed our simulated CD8^+^ T cell to express the same set of surface markers. We initialized naïve CD8^+^ T cells as CD62L^+^, CD27^+^, and KLRG1^−^ (see section 4), and allow daughter cells to noisily inherit the CD62L, CD27, and KLRG1 expression levels from their mother cell. Expression of the markers only changes upon cell division, and the expression levels do not influence the division and death times of the cell. Inheritance of expression levels also involves stochasticity, and the segregation of each marker is independent of the other markers, and the division and death times. The CD62L marker tends to decrease upon cell division, while KLRG1 tends to increase with cell division. By these assumptions, large families tend to be dominated by KLRG1^+^ cells, and contain a small fraction CD62L^+^ cells (Figure 3). Overall, the CD62L marker is negatively correlated (Spearman's ρ ~ − 0.52; *p*-value < 10^−5^) with family size, while the KLRG1 marker is positively correlated (Spearman's ρ ~ 0.73; *p*-value < 10^−5^) (Figures 3A,C). The correlation of CD62L with family size is weaker, and the correlation of KLRG1 is stronger, than the corresponding correlations observed in lineage tracing experiments (8, 10). Corroborating the lineage tracing experiments, CD27 exhibited a weak (Spearman's ρ ~ −0.08) correlation with family size (Figure 3B). Thus, assuming stochastic inheritance of marker expression suffices to explain the previously observed marker dynamics of differentiating CD8^+^ T cells (8, 10).

### 2.3. Heterogenous Time of Activation Model

**Figure 4:**
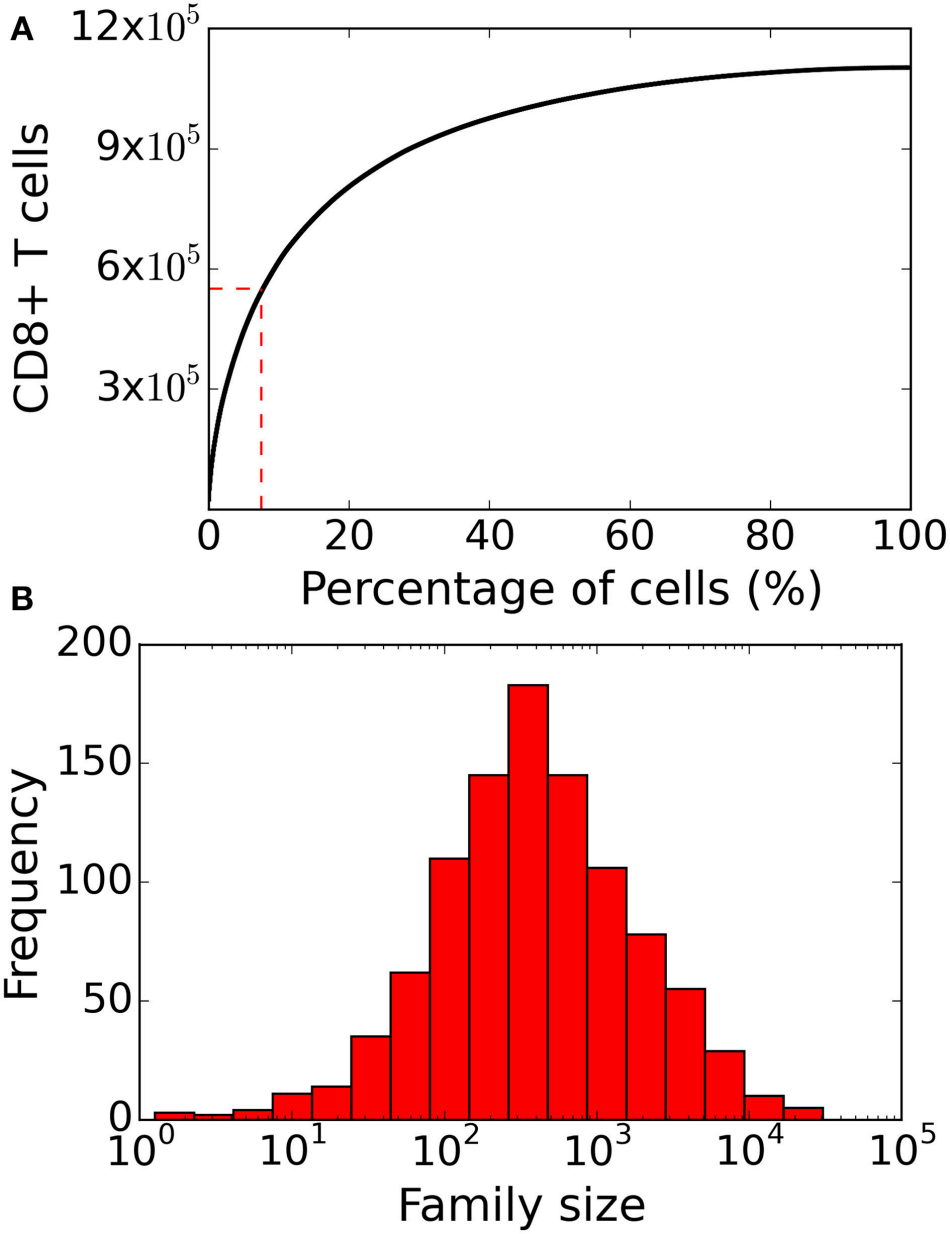
Family size distribution of the heterogenous time of activation model. **(A)** Same as Figure 2 the cumulative family size distribution shows the percentage of naïve CD8^+^ T cells (x-axis) and the cumulative number of responding CD8^+^ T cells present on day 8 (y-axis). The red vertical and horizontal lines represent the fraction of families that constitute 50% of the total response. **(B)** Family size frequency distribution on day 8 shows that some families contribute more than 10^4^ cells to the total response. These results are based on simulating the clonal expansion of a 1,000 naïve CD8^+^ T cells.

In the lineage tracing experiments several small families contained a large fraction of CD62L^+^ cells (8, 10), and a natural explanation for this observation is that these are families completing few divisions because they started late. Although the population dynamics, and the correlations of markers with family size, in our model were comparable to those in these lineage tracing experiments, we rarely observed small CD62L-rich families because in the model small families are largely due to excessive cell death. This is due to one of our simplifying assumptions because in our model the first division tends to be completed faster than the subsequent divisions, whereas in reality the first division typically takes much longer, because quiescent naïve T cells have to become activated and change the expression of thousands of genes. The observed times to complete the first division following activation of naïve CD8^+^ T cells typically obey a normal or lognormal distribution (15, 17, 27, 31, 34). We therefore make our model more realistic by adding a “time of activation” for every naïve CD8^+^ T cell, which is sampled from a lognormal distribution (see section 4 for details). Similar to the basic model, a 1,000 naïve CD8^+^ T cells typically produced >10^6^ CD8^+^ T cells after 8 days of clonal expansion. Adding heterogeneity in the time of activation increased the disparity in the family size distribution (Figure 4), resulting in an even smaller fraction (~7%) of the 1,000 naïve CD8^+^ T cells contributing the majority of the response (Figure 4A). The disparity in the family size distribution in the heterogenous time of activation model more closely resembles the disparity observed in Gerlach et al. (8), and in agreement with their previous interpretation, we conclude that heterogeneity in time of activation plays an important role in T cell population dynamics.

**Figure 5:**
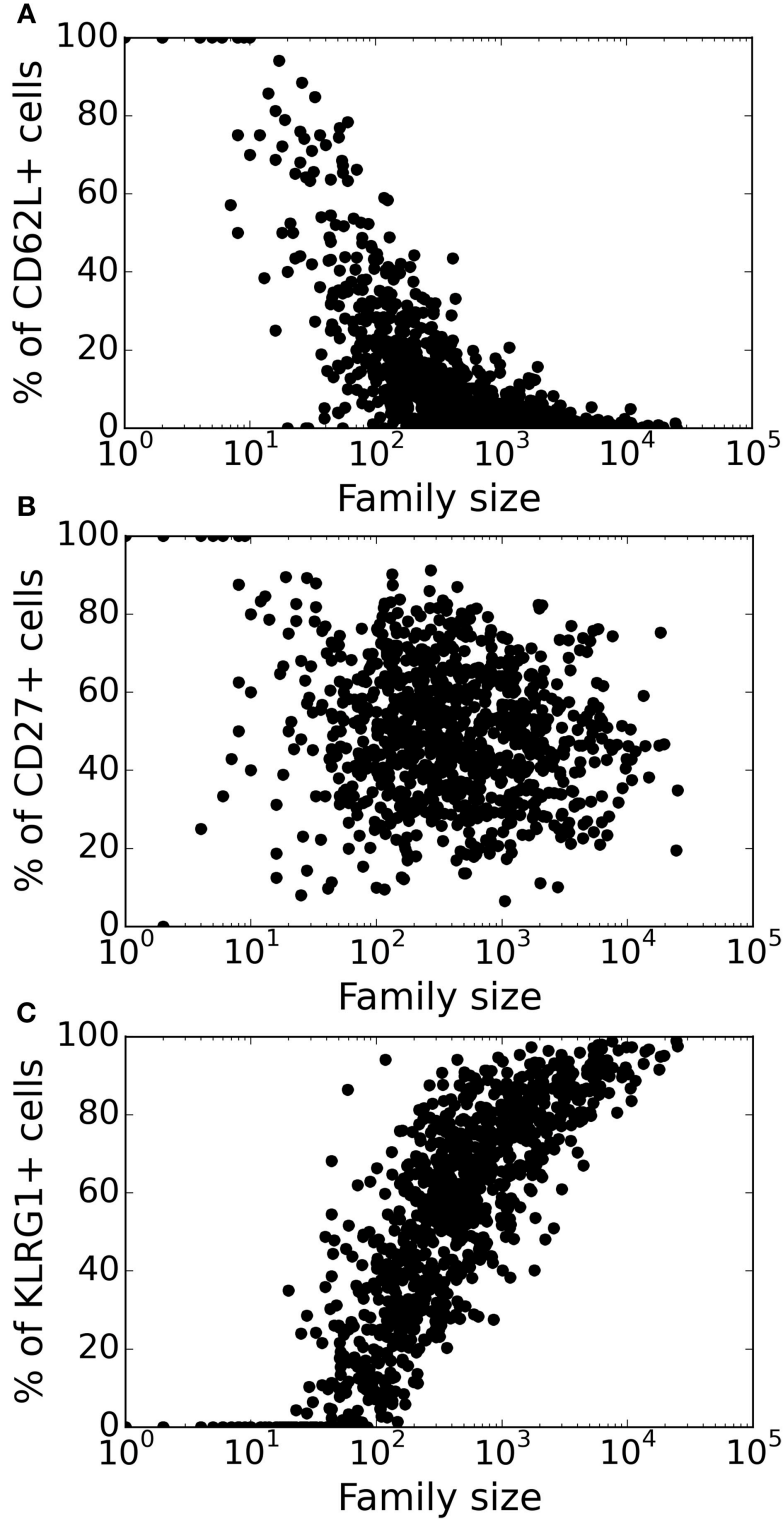
Variation of marker distribution with family size for the heterogenous time of activation model. The fraction of CD8^+^ T cells that are positive for CD62L **(A)**, CD27 **(B)**, and KLRG1 **(C)** markers is shown for each CD8^+^ T cell family. Each bullet represents one naïve CD8^+^ T cell family. These results are based on simulating the clonal expansion of a 1,000 naïve CD8^+^ T cells.

We also studied the marker dynamics in this “heterogenous time of activation” model. Similar to the basic model, in large families a small fraction of cells was CD62L^+^ and most cells were KLRG1^+^ (Figure 5). The CD62L marker was negatively correlated (Spearman's ρ ~ −0.766, *CI*_95%_ = (−0.790, −0.738); Student's *t*-test *p*-value < 10^−10^), while the KLRG1 marker was positively correlated (Spearman's ρ ~ 0.835, *CI*_95%_ = (0.815, 0.853); Student's *t*-test *p*-value < 10^−10^) with family size (Figures 5A,C). CD27 exhibited a weak (Spearman's ρ ~ −0.152) correlation with family size (Figure 3B). These correlations of CD62L, CD27, and KLRG1 are comparable to those observed in the lineage tracing experiments (8, 10), and we now have several small families with a large fraction of CD62L^+^ cells, because some families start late and complete relatively few divisions. Thus, incorporating heterogeneity in time of activation helps to account even better for the marker dynamics of differentiating CD8^+^ T cells (Figure 5).

### 2.4. Division Dependent Marker Dynamics

**Figure 6:**
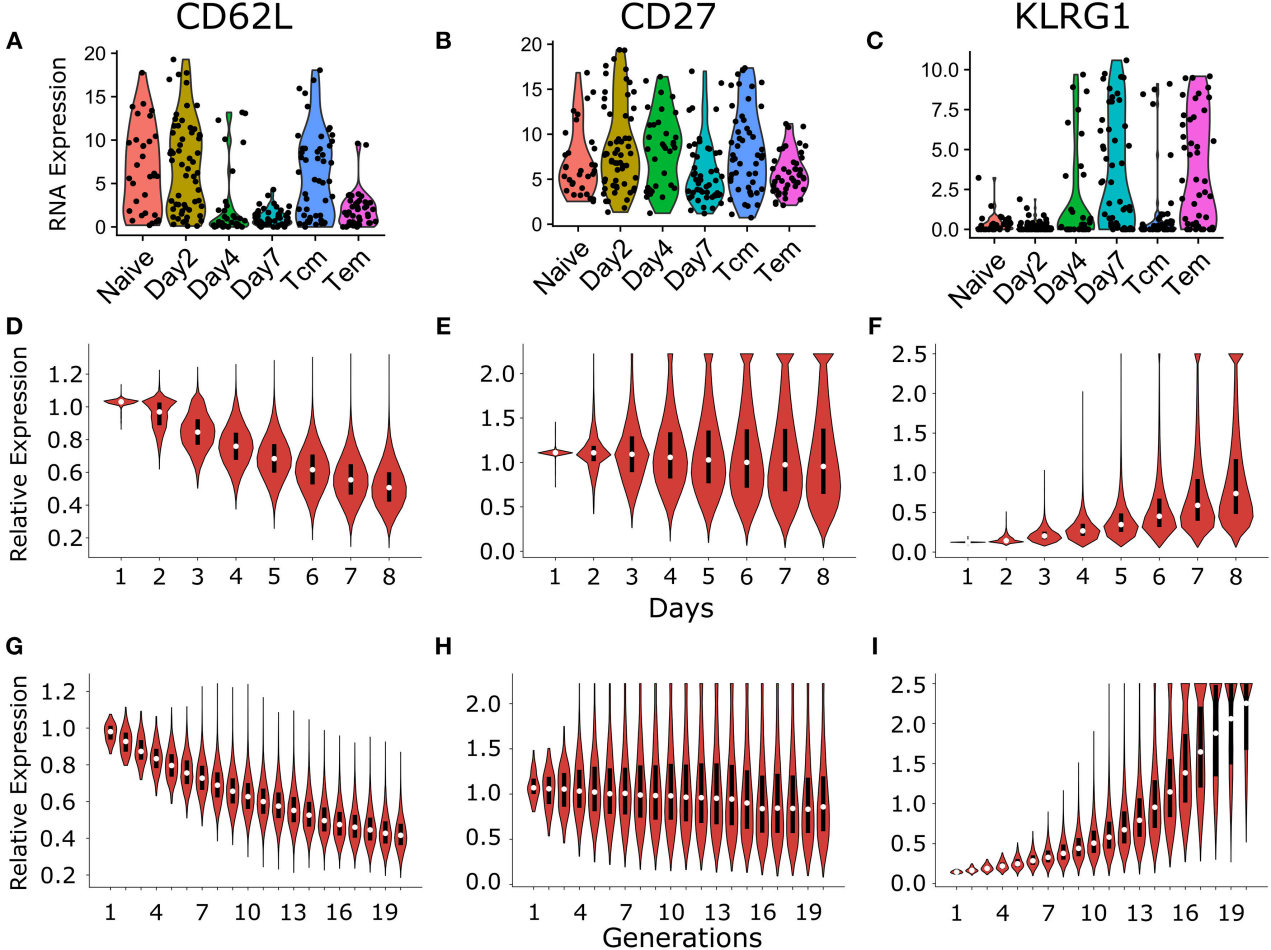
Comparison of temporal and generational dynamics in heterogenous time of activation model with the publicly available single cell experiment described in Kakaradov et al. (26). RNA expression of different cell surface markers CD62L **(A)**, CD27 **(B)**, and KLRG1 **(C)**, which are commonly used to classify different CD8^+^ T cell subsets [reanalyzed from (26)]. Briefly, Kakaradov et al. (26) performed single cell RNA sequencing on the following CD8^+^ T cell populations: naïve; day 2 cells that had undergone their first division (Day2); day 4 cells (Day4); day 7 cells (Day7); day 42 central memory (Tcm); and day 42 effector memory (Tem) cells. The violin plot shows the temporal dynamics of CD62L **(D)**, CD27 **(E)**, and KLRG1 **(F)** expression distribution in CD8^+^ T cells. The cells that were alive at the start of each day were considered for the calculation of the individual expression distribution. The violin plot shows the dynamics of CD62L **(G)**, CD27 **(H)**, and KLRG1 **(I)** expression distribution per generation of CD8^+^ T cells. The marker expression levels in the model were normalized with the expression threshold used to determine marker positivity in Figures 3, 5 (see section 4). These results are based on simulating the clonal expansion of a 1,000 naïve CD8^+^ T cells.

Most studies ascribe the effector and memory potential of dividing CD8^+^ T cells by their surface expression of markers like CD62L, CD27, CCR7, and KLRG1. We reanalyzed the single cell data of proliferating CD8^+^ T cells published by Kakaradov et al. (26) to study the variation in mRNA expression (as an indicator of surface expression of the corresponding proteins). Kakaradov et al. (26) studied single cell mRNA expression data for naïve CD8^+^ T cells (Naive), cells which had undergone 1 division on day 2 (Day2), pools of dividing cells on day 4 (Day4) and day 7 (Day7), central memory (Tcm; CD62L^+^) cells on day 42; and effector memory (Tem; CD62L^−^) cells on day 42 (Figures 6A–D). Note that the last two sets were measured after six weeks, which is beyond the time window of our simulations. Because we are modeling the first 8 days of clonal expansion, the majority of the single cells studied on day 4 and day 7 should be activated effector cells (Figures 6A–C). The expression of CD62L mRNA was high in individual naïve cells, and the Day2 cells undergoing their first division. Since CD62L expression is not dramatically reduced in cells after their first division (Figure 6A), these data confirm that CD62L is lost somewhat gradually. Similarly, the data reveal that CD27 expression does not exhibit a temporal trend (Figure 6B), while KLRG1 mRNA expression increases over the first week (Figure 6C). Thus, single cell expression profiles confirm the assumptions made in our model: the expression of typical markers is gained or lost gradually by dividing CD8^+^ T cells.

Using the heterogenous time of activation model, we explored the longitudinal marker dynamics in dividing CD8^+^ T cells. As expected, the mean CD62L expression decreased over time, but a small fraction of cells remained CD62L^+^ (CD62L expression ≥ marker specific threshold; see section 4) throughout the expansion phase (Figure 6D). The mean CD27 expression was centered around 1 (relative to the marker specific threshold) and exhibited no clear trend (Figure 6E). Nevertheless, several individual CD8^+^ T cells express very low or very high levels of CD27, indicating that stochastic gain or loss of CD27 does skew the marker expression in dividing cells. The mean KLRG1 expression increased over time, however, the majority of the cells remained KLRG1^−^ (KLRG1 expression < marker specific threshold; see section 4) throughout the simulation (Figure 6F).

Two different mechanisms allow dividing cells to maintain high CD62L expression levels (or low KLRG1 expression levels) throughout the expansion phase. First, because of the heterogeneity in the time of first division several families retain high CD62L levels (or low KLRG1 levels) by starting late and completing few divisions. Second, the dividing cells stochastically retain or gain CD62L expression (or retain or lose KLRG1 levels). We therefore plotted the marker distributions as a function of the number of divisions undergone by each cell (Figures 6G–I). As expected cells having undergone few divisions are typically CD62L positive (relative expression ≥1; Figure 6G). Interestingly, a small fraction of cells that had undergone more than 6 divisions were found to be CD62L positive (Figure 6G), indicating that dividing cells can retain or gain CD62L expression due to stochasticity in marker dynamics. The marker distributions of CD27 expression exhibited a slight negative trend with increasing generation number indicating that stochastic retention or acquisition dominates CD27 positivity (Figure 6H). The marker distributions of KLRG1 expression exhibited a strong positive trend with increasing generation number, indicating that most KLRG1 positive cells have undergone a large number of divisions (Figure 6I). A fraction of cells that underwent more than 14 divisions remained KLRG1 negative (relative expression < 1; Figure 6I). Thus, stochasticity in marker inheritance can lead to large variability in marker expression, and classifying CD8^+^ T cells by their markers need not reflect their division history.

### 2.5. T Cell Population and Subset Dynamics

**Figure 7:**
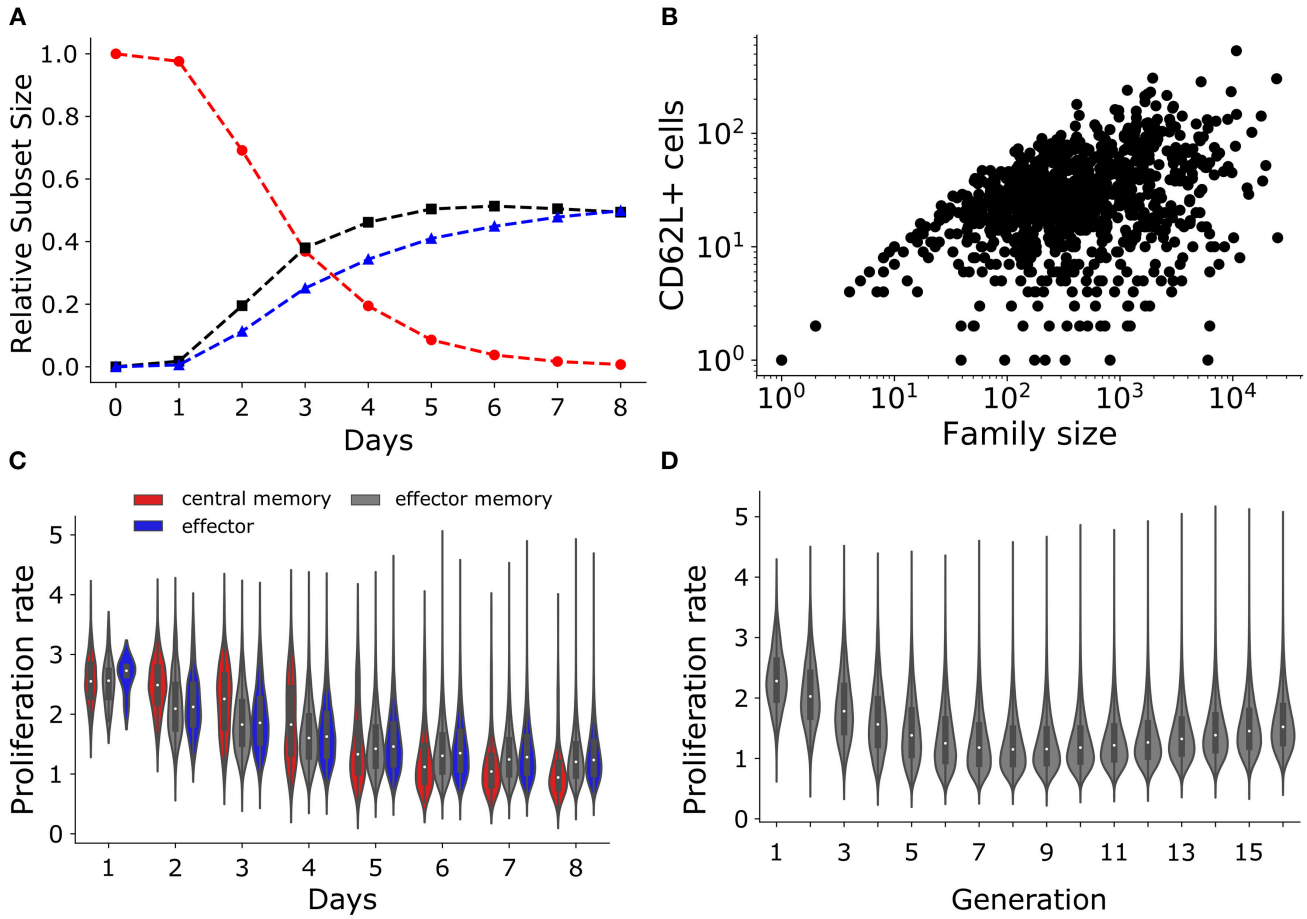
T cell subset dynamics. **(A)** Temporal dynamics of T cells: central memory cells (CD62L^+^CD27^+^; red); effector memory (CD62L^−^CD27^+^; black) cells; and effector (CD62L^−^CD27^−^; blue) cells. **(B)** Number of CD62L^+^ cells as a function of family size. **(C)** The violin plot shows the changes in the rate of proliferation (1/(time of division)) over time for T cell subsets: central memory cells (CD62L^+^CD27^+^; red); effector memory (CD62L^−^CD27^+^; gray) cells; and effector (CD62L^−^CD27^−^; blue) cells. **(D)** The violin plot shows the rate of proliferation as a function of generation or number of divisions.

In a seminal study, Buchholz et al. (10) used lineage tracing and mathematical modeling to suggest that naïve CD8^+^ T cell first divide and differentiate into central memory cells, which further differentiate into effector memory cells, and subsequently into effectors. To mimic their T cell subsets, we similarly categorized the T cells in our simulations: CD62L^+^CD27^+^ as central memory T cells, CD62L^−^CD27^+^ as effector memory T cells, and CD62L^−^CD27^−^ as effector T cells. Lumping our simulated T cells into these subsets our model mimics the population dynamics observed in Buchholz et al. (10) because the sub-population of central memory T cells (red) decreased over time, while the effector memory (black) and effector T cells (blue) increased over time (Figure 7A). Using their progressive model, Buchholz et al. (10) postulated that family sizes are largely determined by the stochastic activation, differentiation and expansion of the cells. Large families arise when activation occurs early and cells differentiate rapidly (as the more differentiated subsets were estimate to proliferate faster). Hence, the memory potential (defined by the expression of the CD62L and CD27 markers) of large families could be lower than that of small families. Although in our model large families tend to produce a small *fraction* of CD62L^+^ “memory” T cells (Figures 3, 5), we found that large families produced the highest *number* of CD62L^+^ “memory” T cells (Figure 7B). Thus, if the expression of CD62L at the end of the expansion phase would indeed correlate with memory potential, e.g., if CD62L^+^ cells were to preferentially survive during the contraction phase, we would conclude that the largest families contribute most to a secondary response [which agrees well with the data of Gerlach et al. (8)].

Using a mathematical model Buchholz et al. (10) inferred that the proliferation rate increases with differentiation, i.e., central memory cells have a lower proliferation rate than effectors. In agreement with this, we found that the proliferation rate (defined as the inverse of the division time) was higher for the effector subset compared to effector memory and central memory subsets when calculated from day 5 onwards (i.e., on day 5 to day 8; Figure 7C). Conversely, the proliferation rate of the central memory and effector memory subset was higher than that of effector subset at early times (i.e., on day 2, 3, and 4 Figure 7C). Finally, we observed that the division rate increased in cells having completed 8 divisions (Figure 7D). Therefore, lineages undergoing a high number of divisions tend to proliferate faster than those undergoing few divisions, which in our model emerges as a consequence of the competition between the division and death rates (Figures 7D, 1B,C), i.e., rapidly dividing families are selected and start to predominate. Hence, large families are formed from cells that tend to divide faster and they produce the highest number of CD62L^+^ “memory” T cells. Thus, the estimated increase in the division rate of clonally expanding CD8^+^ T cell observed by Buchholz et al. (10) could just be a consequence of selection, and need not reflect inherently different kinetic properties of cells adopting different fates defined by the expression of the CD62L and CD27 markers.

## 3. Discussion

How naïve CD8^+^ T cells adopt their eventual fate in terms of memory or effector cells has remained an open question for decades. We have shown that stochastic inheritance of the division and death time of mother cells readily accounts for the large heterogeneity in family sizes that is observed in experimental data (4, 8, 10), and that stochastic inheritance of marker expression can account for the observed kinetic “fates” (10) of cells adopted during the clonal expansion phase. Although the dividing cells in our model do not adopt any fate, as they only gradually change their expected time to divide or die and their marker expression per division, we also obtain that the average proliferation rate of the population increases over time. This is not because the cells adopt different fates but because there is natural selection for families that divide fast, which has the natural side effect that these families tend to be dominated by CD62L^−^ and KLRG1^+^ cells. When the inheritance of the marker expression and the division and death times is noisy, the correlation between the markers and the kinetic properties remains rather poor. Modeling clonal expansion at the single cell level also allowed us to compare the model with the recently published single cell RNA expression data (26), revealing that during the first week of clonal expansion there is little evidence for a separation of the population into clusters of CD62L^+^CD27^+^ central memory T cells, CD62L^−^CD27^+^ effector memory T cells, and CD62L^−^CD27^−^ effector T cells (see Figures 6A–C). This probably happens later, since sorting cells on the basis of these markers on day 42 does lead to subsets with different properties (26). The growth of high-throughput methods therefore warrants the need to develop models incorporating the dynamics of individual cells.

Studies have shown that gain or loss of surface markers is a continuous process (11, 25, 26, 33). Nevertheless, several models categorize dividing T cells into distinct sub-populations having different properties (10, 26, 35). Although our model shows that a large fraction of cells undergoing a high number of divisions tend to lose CD62L, and that these cells tend to divide faster, we also found that a fraction of cells can retain or gain CD62L expression, despite undergoing several divisions. Similarly, we found a substantial fraction of cells can remain KLRG1 negative after undergoing a high number of divisions. Thus, categorizing dividing T cells into subsets using the expression of a few surface marker need not reflect the true dynamics of individual CD8^+^ T cells. More importantly, Buchholz et al. (10) categorized dividing T cells into subsets and postulated that larger families produce smaller fraction of CD62L^+^ cells at the end of the expansion phase. Using their mathematical model Buchholz et al. (10), predict that the contribution to memory phase is poorly correlated with the size of a family. Contrary to this, Gerlach et al. (8) reported that the contribution to the memory phase is positively correlated with the size of a family at the end of the expansion phase. This is the expected result when the activated cells at the end of the expansion phase do not differ much in their survival probabilities during the subsequent contraction phase. Additionally, if the expression of CD62L at the end of the expansion phase were to correlate with memory potential, i.e., if CD62L^+^ cells were to preferentially survive during the contraction phase, our model would still predict that the largest families contribute most to a secondary response, because they contain more CD62L^+^ cells than smaller families (Figure 7B), even though the latter contain a larger *fraction* of CD62L^+^ cells (Figure 5A).

**Figure 8:**
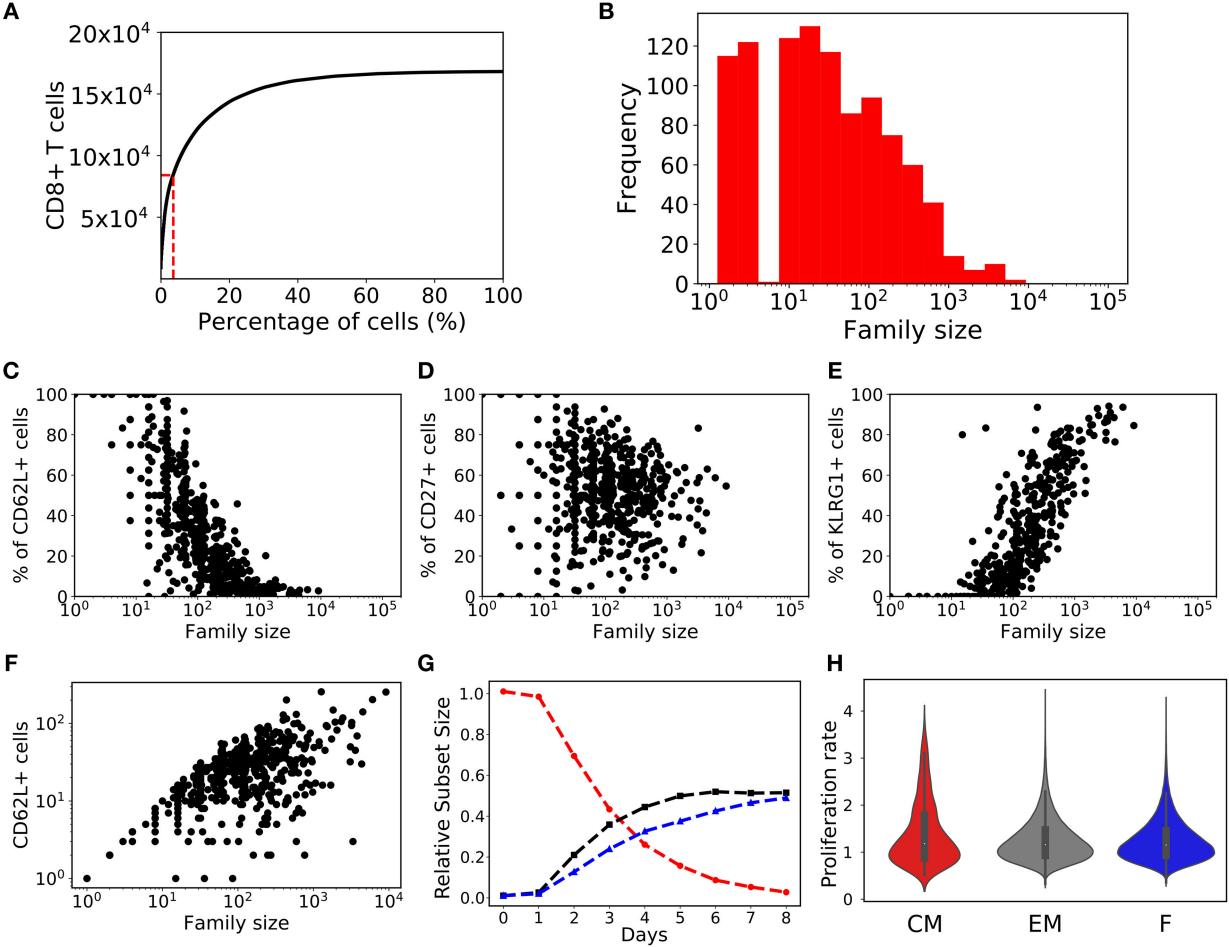
Division destiny stochastic inheritance model. **(A)** Cumulative family size distribution, and **(B)** family size frequency distribution on day 8 from division destiny stochastic inheritance model (same as Figure 2). The fraction of CD8^+^ T cells that are positive for CD62L **(C)**, CD27 **(D)**, and KLRG1 **(E)** markers is shown for each CD8^+^ T cell family (same as Figure 3). **(F)** Number of CD62L^+^ cells as a function of family size (similar to Figure 7B). **(G)** Temporal dynamics of T cells (similar to Figure 7A). **(H)** The violin plot shows the rate of proliferation (1/(time of division)) for T cell subsets from day 5 to day 8: central memory cells (CD62L^+^CD27^+^; red); effector memory (CD62L^−^CD27^+^; gray) cells; and effector (CD62L^−^CD27^−^; blue) cells (similar to Figure 7C).

Several studies have indicated that the division times of two daughter cells are highly correlated, and that the *division destiny* (number of divisions after activation) of a naïve T cell is determined by integrating the activation and co-stimulatory signals (16, 17, 36). Myc was identified as regulator of *division destiny* with the rate of production of Myc being inherited from one generation to the next. Interestingly, the time of death was not affected by Myc expression; hence time of division and death are regulated independently (16, 17). Allowing for stochasticity in the inheritance of division and death times in our models implicitly defines different division destinies for different families. To test whether or not defining an explicit division destiny (i.e., a maximum number of generations) for each family would change our conclusions, we extended our heterogeneous time of activation model with a phenomenological parameter sampling the maximum number of divisions for each family from a lognormal distribution (see Equation 7). Cells breaching their predetermined division destiny are assumed to become quiescent. We found that stochastic inheritance model with division destinies exhibited population dynamics (Figures 8A,B), marker dynamics (Figures 8C-F), and subset based inferences (Figures 8G,H) that were qualitatively similar to the stochastic inheritance model without division destinies. We found that incorporating division destinies increased the skewness in the response (fewer families were required to mount 50% of the response) as a large number of families with rapidly dividing cells reached their division destinies curtailing their response potential (Figure 8B). Varying the distribution from which we sampled the division destinies changed the skewness of the responses (data not shown), but did not alter our qualitative results and inferences.

In conclusion, we show that modeling stochastic inheritance of time of division and death allows us to qualitatively reproduce the marker, division and differentiation dynamics of CD8^+^ T cells without having to assume that clonally expanding cells adopt different fates.

## 4. Methods

### 4.1. Basic Model

Upon activation by cognate antigen, naïve T cells clones undergo rampant proliferation to produce a large population of effector and memory T cells. We considered a modified cyton model (27) that we call the *stochastic inheritance model* to capture the proliferation and differentiation of naïve CD8^+^ T cells. Similar to cells in the Cyton model, each cell in our model harbors two competing clocks: (i) a *division clock* which determines the time of division, and (ii) a *death clock* which determines the time of death (27, 28, 37, 38). If the division time is smaller than the death time, the cell divides at the time of division; whereas if the death time is smaller than the division time, the cell dies at the time of death. We simplified extensions of the Cyton model that allow each daughter cell to stochastically inherits the division and death times from its mother (27–30, 37). In our model the daughters inherit the division and death times by sampling from a lognormal distribution. The time of the first division of each naïve T cell is sampled from a lognormal distribution as follows:

(1) $$t_{p_{0}}\;=\;L(\mu_{mp},v_{mp}),$$

where *t*_*p*_0__ is the *t*ime at which a cell in generation 0 *p*roliferates. The division time of the daughters in generation *i* is inherited from their mother cell in generation *i* − 1 by sampling from another lognormal distribution:

(2) $$\begin{array}{ll} t_{p_{i}} & =t_{p_{i-1}}\times L(\mu_{p},v_{p}), \end{array}$$

where, *i* is the generation, *t*_*p*_*i*−1__ is the division time of the mother cell. We assumed that the division times tend to increase with every division and therefore set *μ*_*p*_ > 1 (Table 1). Additionally, we required a minimum division time of *t*_*min*_ = 4 hours for every division (Table 1), and redraw *t*_*p*_*i*__ whenever *t*_*p*_*i*__< *t*_*min*_ (for *i* ≥ 0, i.e., including the first division). We tested different *μ*_*p*_ values and found no qualitative difference between our results (Figure S1 in Supplementary Material). Since stochastic factors may lead to variation between the division times of the daughter cells (*t*_*p*_*i*, 1__ and *t*_*p*_*i*, 2__), we considered an additional variation in the division times of each daughter,

(3) $$t_{p_{i,1}}=t_{p_{i}}\times N(1,v_{e})\text{ and }t_{p_{i,2}}=t_{p_{i}}\times N(1,v_{e}),$$

by sampling twice from a normal distribution $N\left(1,v_{e}\right)$ (Table 1).

Similarly, we defined the time of death for each naïve T cell, *t*_*d*_0__, and each daughter cell, *t*_*d*_*i, j*__, using lognormal distributions,

(4) $$t_{d_{0}}=L(\mu_{md},v_{md}),$$

(5) $$t_{d_{i,1}}=t_{d_{i-1}}\times L(\mu_{d},v_{d}),\text{ and }t_{d_{i,2}}=t_{d_{i-1}}\times L(\mu_{d},v_{d}),$$

where we set *μ*_*d*_ < 1 to define that cells tend to shorten their time to death with every division.

### 4.2. Heterogenous Time of Activation Model

In the *basic model* the time the naïve mother cells take to complete their first division is very similar to the time required to complete the subsequent divisions. Several studies have shown that the time to complete the first division is much longer than that of subsequent divisions (15, 27, 28), which is natural because quiescent naïve T cells have to become activated and change the expression of thousands of genes. To allow for a difference between the first and the subsequent divisions we add a stochastic “activation time” to the first division. Activation is expected to be stochastic because of intrinsic differences among the naïve T cells and their environments. Adding an activation time of on average *μ*_*a*_ hours, we define the time of first division as a sum of two lognormal distributions:

(6) $$t_{p_{0}}\;=L(\mu_{mp},v_{mp})+L(\mu_{a},v_{a}),$$

where we use an average time of activation of 2 days, i.e., *μ*_*a*_ = 57 and *v*_*a*_ = 29 (15).

### 4.3. Division Destiny Model

To incorporate an explicit division destiny, we sample a maximum number of divisions allowed for each family from a lognormal distribution as:

(7) $$D_{f}\;=L(\mu_{div},v_{div}),$$

where the *div*ision destiny of a family, *D*_*f*_, defines the highest generation number allowed within a family *f*. We parameterized the model such that the mean division destiny was 8 generations (i.e., *μ*_*div*_ = 1.73 and *v*_*div*_ = 0.83). When cells breach their division destiny they become quiescent, and do not undergo further divisions nor cell death.

### 4.4. Marker Inheritance

In the simulations, every naïve CD8^+^ T cell was seeded with three initial expression levels of the surface markers CD62L, CD27, and KLRG1 by sampling normal distributions:

(8) $$CD62L_{0}=N(100,0.01)$$

(9) $$CD27_{0}\;=N(100,0.01)$$

(10) $$KLRG1_{0}\;=N(10,0.01)$$

where the index 0 again indicates the expression level in the naïve mother cells (generation 0), and we use arbitrary levels, i.e., 100 for high and 10 for low.

Upon division the three markers are stochastically inherited by the daughter cells such that the CD62L marker tends to decrease, while the KLRG1 marker tends to increase upon cellular division. We considered that CD27 marker was stochastically inherited without any propensity to increase or decrease upon cellular division, i.e.,

(11) $$\begin{array}{l} CD62L_{i,1}\;=CD62L_{i-1}\times N(0.95,0.05)\text{ and }CD62L_{i,2}\\ \;=CD62L_{i-1}\times N(0.95,0.05), \end{array}$$

(12) $$\begin{array}{l} CD27_{i,1}\;=\;CD27_{i-1}\times N(1.0,0.15)\text{ and }CD27_{i,2}\\ \;=CD27_{i-1}\times N(1.0,0.15), \end{array}$$

(13) $$\begin{array}{l} KLRG1_{i,1}\;=\;KLRG1_{i-1}\times N(1.15,0.15)\text{ and }KLRG1_{i,2}\\ \;=KLRG1_{i-1}\times N(1.15,0.15), \end{array}$$

where, *CD*62*L*_*i, j*_, *CD*27_*i, j*_, and *KLRG*1_*i, j*_ are the expression levels of the CD62L, CD27, and KLRG1 markers for each daughter cell (*j* = 1 or 2), which are inherited from the expression level in their respective mother cells. Note that CD62L expression tends to decrease by 5% per division, and that KLRG1 expression tends to increase by 15%. For Figures 3, 5–8, we considered cells with a CD62L expression ≥ 97, CD27 expression ≥ 90, and KLRG1 expression ≥ 80 as positive, for the respective markers. Since expression levels are arbitrary, we also considered arbitrary thresholds to assign marker positivity. Due to lack of data, these propensities had to be guessed, but changing them produced qualitatively similar results, albeit with higher or lower variability in the marker distributions. Also note that in our models the expression levels can only reflect the division history of a cell and that they do not change the behavior of the cells. Since the cells in our model cannot adopt memory or effector fates, the CD62L, CD27, and KLRG1 expression cannot reflect these fates.

The codes for simulating the three models are available at: https://bitbucket.org/aridaman/tcellfate.

### 4.5. Single Cell Data Analysis

Single cell expression data described and generated by (26) was downloaded from NCBI GEO website (Accession number: GSE89405). We used R package Seurat (39) to normalize and quality check the single cell data. The gene expression for selected genes were plotted using R package ggplot2 (40).

## Author Contributions

AP and RD conceptualized and designed the study, and interpreted the results. AP performed the analysis. Both authors contributed to writing the manuscript, read and approved the submitted version.

### Conflict of Interest Statement

The authors declare that the research was conducted in the absence of any commercial or financial relationships that could be construed as a potential conflict of interest.
