# Supplementary material for: Stochastic Inheritance of Division and Death Times Determines the Size and Phenotype of CD8+ T Cell Families
Source: Front Immunol. 2019 Mar 14;10:436. doi: 10.3389/fimmu.2019.00436 (PMC6426761; doi:10.3389/fimmu.2019.00436)
Supplement: Supplementary file 1 [file Data_Sheet_1.PDF]

frontiers

Supplementary Material:
Stochastic inheritance of division and death times determines the size and phenotype of CD8$^+$ T cell families

1 SUPPLEMENTARY FIGURE

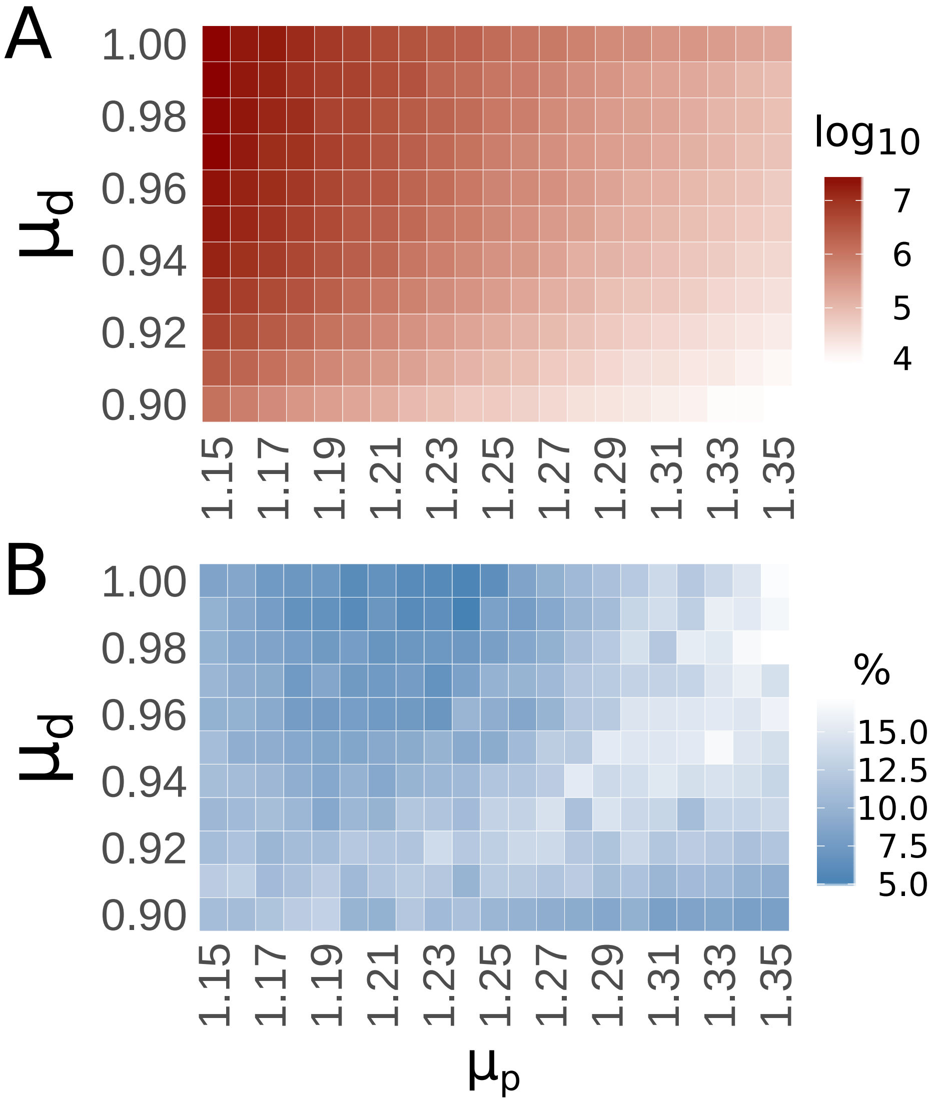

Figure S1. Parameter sweep. Panel A shows the total CD8$^+$ T cell population sizes and Panel B shows the fraction of families that constitute 50% of the total response on day 8, for different values of μ_p and μ_d. In Panel A, the red color depicts the log_{10} population sizes, such that the darker the color the large the population size. In Panel B, the blue color depicts the percentage of families constituting 50% of the total response, such that the lighter the color the higher the percentage. These results are based on simulating the clonal expansion of a 1000 naïve CD8$^+$ T cells for each parameter setting.
